# Antifungal Mechanisms of a Chinese Herbal Medicine, Cao Huang Gui Xiang, Against *Candida* Species

**DOI:** 10.3389/fphar.2022.813818

**Published:** 2022-03-09

**Authors:** Huizhen Yue, Xiaolong Xu, Shasha He, Xuran Cui, Yuhong Guo, Jingxia Zhao, Bing Peng, Qingquan Liu

**Affiliations:** ^1^ Beijing Hospital of Traditional Chinese Medicine, Capital Medical University, Beijing, China; ^2^ Beijing Institute of Chinese Medicine, Beijing, China; ^3^ Beijing Key Laboratory of Basic Research with Traditional Chinese Medicine on Infectious Diseases, Beijing, China

**Keywords:** Cao Huang Gui Xiang formula, *Candida albicans*, antifungal activity, biofilm formation, ROS production, Ras1-cAMP pathway

## Abstract

Cao Huang Gui Xiang (CHGX) formula, a Chinese herbal medicine, has been empirically used for the treatment of *Candida* infections. In the present study, we discovered that the CHGX showed potent antifungal activities against the major human fungal pathogen *Candida albicans* and other clinical *Candida* species. Besides, we indicated that CHGX had *in vivo* efficacy on treating *C. albicans* infection in mice without noticeable toxicity at the clinical therapeutic concentration. We then set out to investigate the antifungal mechanisms of CHGX against *C. albicans*. We found that CHGX played an important role in inhibiting biofilm formation and filament development, two critical virulence factors of *C. albicans*. We further demonstrated that CHGX disrupted cell membrane integrity, triggered the accumulation of reactive oxygen species (ROS) and consumption of adenosine triphosphate (ATP), followed by a rapid fungal cell death in *C. albicans*. Multiple pathways, including the conserved Ras1-cAMP pathway and mitochondrial protein Mcu1 are involved in CHGX-induced cell death. Our finding expands the understanding of antifungal mechanism of CHGX against *C. albicans*, and provides new insights in treating patients with *Candida* infections in clinical practice.

## Introduction

*Candida albicans* is an opportunistic human pathogen, which can reside as a commensal microbiota in human oral cavity, intestinal tract, and vaginal area. It causes both superficial diseases and life-threatening disseminated infections when patients are exposed to long-term broad-spectrum antibiotics, chemotherapies or are in the immune compromised stage (Brown et al., 2012). Approximately, 50% of cases of *Candida* sepsis were related to invasive *C. albicans* infections (Pfaller and Diekema, 2010; Brown et al., 2012). In recent decades, a noticeable increase has been reported in the incidence of invasive candidiasis, accompanied with a mortality rate of greater than 40% (Kullberg and Arendrup, 2015). This can be highly attributed to the drug resistance to the limited conventional antifungal agents, such as azoles and polyenes, commonly used for treating candidiasis, as well as to the adverse events, such as nephrotoxicity and liver toxicity (Tobudic et al., 2010; Cowen et al., 2014; Sandai et al., 2016). Thus, there is an urgent need to explore novel antifungal agents that are highly efficient, safe, and cost-effective.

Natural products (e.g., extracts and bioactive compounds from plant resources) are regarded as goldmine for the discovery of novel drugs. Medicinal plants from orders of *Acorales* Mart., *Apiales* Nakai, *Asterales* Link, *Lamiales* Bromhead, *Laurales* Juss. ex Bercht. and J. Presl, *Myrtales* Juss. ex Bercht. and J. Presl, *Poales* Small and *Sapindales* Juss. ex Bercht. and J. Presl have exhibited a strong anti-biofilm activity against *Candida* species (Singla and Dubey, 2019). Among them, the extracts from *Solidago virgaurea* L. [Asteraceae] (Chevalier et al., 2012), *Thymbra capitata* (L.) Cav. [Lamiaceae] (Palmeira-de-Oliveira et al., 2012), *Coriandrum sativum* L. [Apiaceae] (Freires Ide et al., 2014), *Peganum harmala* L. [Nitrariaceae] (Aboualigalehdari et al., 2016), and *Syzygium aromaticum* (L.) Merr. and L. M. Perry (Khan and Ahmad, 2012) were found to play significant roles in inhibiting the biofilm formation of *C. albicans* and non-*C. albican*s. Cao Huang Gui Xiang (CHGX) formula is an effective prescription designed for treatment of critically ill patients with oral candidiasis or systemic *Candida* infections by Professor Qingquan Liu, a leading researcher in therapy of septic patients using traditional Chinese medicine (TCM). It has been used for clinical treatment of *Candida* related infections for more than 20 years and exhibited favorable effect in Beijing Hospital of Traditional Chinese Medicine (Beijing, China). As a TCM compound with a complex composition, it is essential to figure out its pharmacological effects and mechanisms against *Candida* infections.

Therefore, the present study aimed to validate the anti-*Candida* properties of CHGX *in vitro* and *in vivo*. Moreover, the effects of CHGX on virulence factors of *C. albicans* were assessed, including the biofilm formation and morphological transition. Importantly, we further explored the mechanisms of action underlying the potential antifungal effects, and evaluated the role of conserved Ras1-cAMP pathway and mitochondrial protein Mcu1 in CHGX-induced cell death in *C. albicans* by regulating reactive oxygen species (ROS) production and adenosine triphosphate (ATP) consumption.

## Materials and Methods

### Strains and Cultivation

The strains and primers used in the current study are listed in Supplementary Tables S2, S3. A yeast extract peptone dextrose (YPD) medium (2% glucose, 2% peptone, and 1% yeast extract) and completely synthetic Lee’s glucose medium were used for the routine growth of fungal yeast cells. YPD medium containing 10% fetal bovine serum (FBS) and RPMI 1640 medium were used for the induction of filamentation, and Spider medium was utilized for the induction of biofilm formation in *C. albicans*.

### Preparation of CHGX

CHGX formula, consists of the root and rhizomes of *Glycyrrhiza uralensis* Fisch. ex DC. [Fabaceae] (Gan Cao, 15 g), the root and rhizomes of *Rheum palmatum* L. [Polygonaceae] (Da Huang, 10 g), the bark of *Neolitsea cassia* (L.) Kosterm. [Lauraceae] (Rou Gui, 10 g), and the aerial part of *Pogostemon cablin* (Blanco) Benth (Guang Huoxiang, 15 g), was provided by Beijing Hospital of Traditional Chinese Medicine (Beijing, China). The ratio of the above four herbs is 3:2:2:3 and the clinical dosage of CHGX is 50 g of crude drug in adults daily based on the long-term clinical practice. Briefly, 100 g crude drug of CHGX was soaked for 30 min, decocted for 30 min in 600 ml deionized water, and collected the first water decoction. The residual drug was decocted again for 20 min in 400 ml deionized water, and collected the second water decoction. The total combined water decoction was concentrated to 100 ml (1 g crude drug/ml, 100%, v/v) as CHGX water-decoction. Then the liquid CHGX water-decoction was freeze-dried into a dark brown mass with the yield of 16% by vacuum, referred to as CHGX water-extract, for the convenience of use in this study.

### Herbs

*Glycyrrhiza uralensis* Fisch. ex DC. [Fabaceae] was purchased from Beijing KangYuanXiangRui Pharmaceutical Technology Co., LTD. (Gansu, China, Voucher number 211209002). *Rheum palmatum* L. [Polygonaceae] was purchased from Beijing ShengShiLong pharmaceutical Co., LTD. (Qinghai, China, Voucher number 2103137). *Neolitsea cassia* (L.) Kosterm. [Lauraceae] was purchased from Beijing Xinglin pharmaceutical Co., LTD. (Guangxi, China, Voucher number 21081303). *Pogostemon cablin* (Blanco) Benth was purchased from Beijing ShengShiLong pharmaceutical Co., LTD. (Guangdong, China, Voucher number 2108079).

### Ultra-Performance Liquid Chromatography-Tandem Mass Spectrometry of CHGX

An UPLC–MS/MS system (Waters Corp., Milford, MA, United States) equipped with a HESI-II probe was utilized to identify the main components of CHGX, as previously reported (Xu et al., 2018). The positive and negative HESI-II voltages were set to 3.8 and 3.2 kV, respectively, and the vaporizer temperature was set to 300°C. Both the sheath gas and the auxiliary gas were nitrogen. The collision gas was also nitrogen at a pressure of 1.5 mTorr. The mobile phase was composed of A (0.1% (v/v) formic acid aqueous solution) and B (acetonitrile). The HPLC elution conditions were optimized as follows: 0–1 min: 95% A; 1–2.4 min: 95–90% A; 2.4–13.5 min: 90–68% A; 13.5–18.5 min: 68–10% A; 18.5–19 min: 10–95% A; and 19–21 min: 95% A. The flow rate and the column temperature were set to 0.3 ml/min and 35°C, respectively. Data were collected and processed by Xcalibur 4.2 software (Waters Corp.).

### Testing of Antifungal Susceptibility

The minimum inhibitory concentration (MIC) was determined according to the NCCLS document M27-A2 and a previous research (Bing et al., 2020). *C. albicans* strain SC5314 was used. In brief, *C. albicans* cells were initially cultured in YPD medium plates at 37°C for 24 h. The colonies were collected and washed twice with ddH_2_O, and then cells of SC5314 were adjusted to 5 × 10^3^ cells/ml in RPMI1640 medium (w/v, 1.04% RPMI-1640, 3.45% MOPs, NaOH used for pH adjustment to 7.0). Approximately 500 fungal cells in 0.1 ml RPMI 1640 medium were mixed with 0.1 ml RPMI 1640 medium with a serial two-fold concentration of CHGX formula in a 96-well plate for MIC assay. Cells were incubated at 37°C for 24 h. Amphotericin B (AMPB) was used as a positive control. *Candida krusei* ATCC 6258 and *Candida parapsilosis* ATCC 22019 served as quality controls.

### The Time-Kill Kinetics Assay of Fungal Cells

Fungal cells from a single colony were initially inoculated in liquid YPD medium and cultured to logarithmic phase at 30°C with shaking. Fungal cells were collected, washed, and adjusted to 2 × 10^5^ cells/ml in Lee’s glucose medium or YPD medium. Cells were then treated with different concentrations of CHGX water-extract at 30 or 37°C. The CUFs of viable cells were determined using plating assays at time points indicated. At least three independent biological replicates of the experiment were conducted, and the proportion of viable cells was calculated as follows: = (CFUs at an indicated time point/CFUs at 0 h) ×100%. The killing activity was calculated from treated as well as untreated cells based on the CFUs count.

### Inhibition of Yeast-To-Hyphal Transition

*C. albicans* yeast cells were initially cultured in liquid YPD medium at a logarithmic phase, and were then harvested, washed, and re-suspended in liquid Lee’s glucose medium. *C. albicans* strain SC5314 was used. 2 × 10^6^ cells/ml cells of SC5314 were treated with different concentrations of CHGX water-extract (0, 10, and 20 mg/ml) in Lee’s glucose medium supplemented with 10% FBS or in RMPI1640 medium at 37°C. The cellular morphology was observed microscopically at 0, 3, 6, 12, or 24 h. The percentage of hyphal cells was expressed as mean ± standard deviation (SD) of three independent experiments.

### Effects of CHGX Water-Extract on Biofilm Formation

The microtiter plate biofilm assay was performed according to a previous research with a slight modification (Du et al., 2012). *C. albicans* strain SC5314 was used. *C. albicans* cells were initially cultured in liquid YPD medium at a logarithmic phase, and were then harvested, washed, and re-suspended in liquid Spider medium. The MIC of CHGX water-extract for biofilm formation of SC5314 in a flat-bottom 96-well plate was determined by the micro-dilution method. Besides, 1 × 10^5^ cells of SC5314 were incubated with different concentrations of CHGX water-extract (20–0.04 mg/ml) in 200 μl Spider medium per well for 3 days at 37°C. The plates were imaged before and after gently washed with phosphate-buffered saline (PBS). After gently washed, the cells adhered to the bottoms of 96-well plates were treated with 10% trypsin in 200 μl PBS and washed twice with 200 μl PBS. The total cells suspension (600 μl/per well) was collected and the cells intensity (OD600) was read with Synergy 4 Gene 5 plate reader (Biotek, Potton, United Kingdom) for quantitation. Three independent biological replicates of the experiment were conducted.

### Propidium Iodide Staining Assay

*C. albicans* cells were initially cultured in liquid YPD medium at a logarithmic phase, and were then harvested, washed, and re-suspended in liquid Lee’s glucose medium. *C. albicans* strain SC5314 was used. 2 × 10^6^ cells/ml cells of SC5314 were treated with or without 20 mg/ml CHGX water-extract in Lee’s glucose medium for 3 h at 30°C, and cells treated with 10 μg/ml AMPB were served as a positive control. Cells were harvested, washed with PBS, and then stained with 5 μg/ml PI for 1 h at 4°C in dark. To perform flow cytometry using a FACS Calibur system (BD Biosciences, San Jose, CA, United States), at least 20,000 events per sample were recorded and analyzed by FlowJo 7.6 software (FlowJo, Ashland, NC, United States). A fluorescence microscope (Carl Zeiss, Berlin, Germany) was used to visualize *C. albicans* cells that were stained with PI. The percentage of PI-stained cells was presented as mean ± SD of three independent experiments.

### Measurement of Intracellular ROS

The intracellular ROS of *C. albicans* was determined using a ROS Assay kit (Beyotime, Shanghai, China), as reported previously (Gong et al., 2019). *C. albicans* strain SC5314 was used. Fungal cells were initially cultured in liquid YPD medium at a logarithmic phase, and were then harvested, washed, and re-suspended in liquid Lee’s glucose medium. *C. albicans* cells (2 × 10^6^ cells/ml) were treated with or without 20 mg/ml CHGX water-extract in Lee’s glucose medium for 3 h at 30°C. Cells were harvested, washed with PBS, and incubated with DCFH-DA probe for 30 min at 37°C in dark. The cells were subjected to flow cytometry using a FACS Calibur system (BD Biosciences) or visualized by a fluorescence microscope (Carl Zeiss). The percentage of stained cells was presented as mean ± SD of three independent experiments.

### Measurement of Intracellular ATP

Intracellular ATP level in *C. albicans* cells was determined using an ATP Assay System Bioluminescence Detection kit (Cat. No. FF2000; Promega Inc., Madison, WI, United States), as reported previously (Tao et al., 2017). Briefly, *C. albicans* yeast (SC5314) cells were initially cultured in liquid YPD medium at a logarithmic phase, and were then harvested, washed, and re-suspended in liquid Lee’s glucose medium. 2 × 10^6^ cells/ml cells of SC5314 were treated with or without 20 mg/ml CHGX water-extract for 3 h at 30°C. Cells were harvested and washed with PBS, and then homogenized in PBS with a bead beater. A representative ATP standard curve was plotted using ten-fold dilutions of ATP. The results were normalized to the corresponding protein concentrations, as determined by the Bradford assay (Bio-Rad Laboratories Inc., Hercules, CA, United States). The values were presented as mean ± SD of three independent experiments.

### Transmission Electron Microscopy

Transmission Electron Microscopy (TEM) was undertaken according to a previously reported protocol (Wright, 2000). Firstly, *C. albicans* yeast (SC5314) cells were initially cultured in liquid YPD medium at a logarithmic phase, and were then harvested, washed, and re-suspended in liquid Lee’s glucose medium. 2 × 10^6^ cells/ml cells of SC5314 were treated with or without 20 mg/ml CHGX water-extract, and then were used for TEM. Cells were fixed with 2.5% glutaraldehyde and 0.5% polyoxymethylene in a buffer solution (0.2 M PIPES, pH 6.8; 1 mM MgCl2; 1 mM CaCl2; 0.1 M sorbitol), and then washed four times with deionized water. Cells were stained with 1.5% KMnO_4_ for 1 h and subjected to dehydration using concentrated cold acetone solutions (50, 75, 85, 95, 100, 100, and 100%). Cells were subsequently infiltrated and embedded into Spurr resin for TEM (JEM-1400; JEOL, Tokyo, Japan).

### RNA Extraction and Quantitative RT-PCR Assay

RNA extraction and qRT-PCR assay were performed, as described previously with a modification (Yue et al., 2018). Firstly, *C. albicans* yeast (SC5314) cells were initially cultured in liquid YPD medium at a logarithmic phase, and were then harvested, washed, and re-suspended in liquid Lee’s glucose medium. 2 × 10^6^ cells/ml cells of SC5314 were treated with or without 20 mg/ml CHGX water-extract in Lee’s glucose medium at 30°C for 3 h. Cells were harvested for extraction of total RNA using the GeneJET RNA purification kit (Thermo Fisher Scientific, Waltham, MA, United States) according to the manufacturer’s instructions. For qRT-PCR assay, 1 μg of total RNA per sample was used to synthesize cDNA using the RevertAid Reverse Transcriptase kit (Thermo Fisher Scientific). Quantification of transcripts was carried out using a Bio-Rad CFX96 real-time PCR detection system with a SYBR green Mix kit (Toyobo Co., Ltd., Tokyo, Japan). The expression level of each protein was normalized to that of *CaACT1*.

### Animal and Ethic Statement

BALB/c mice (female, 15–18 g, 5 weeks old) were purchased from SiBeiFu Bioscience Co., Ltd. (Beijing, China). Animals were housed in a BSL2 barrier animal facility. All animal experimental procedures described here were conformed to the Guide for the Care and Use of Laboratory Animals published by the US National Institutes of Health, Publication No. 8023, revised 1978. In addition, all protocols in this study were approved by the permission of Institute of China Academy of Chinese Medical Sciences, Chinese Materia Medica, Ethic Committee.

### *In vivo* Toxicity Evaluation

Animals were divided into a control group and two experiment groups (Low-dose, High-dose) according to the CHGX water-decoction dosage. There were five mice in each group. Mice in experiment groups were orally administered with CHGX water-decoction at doses of 6.5 g/kg/day and 13 g/kg/day, namely low- and high-dose groups, respectively. The low-dosage of CHGX water-decoction corresponded to the clinical dosage of CHGX water-decoction in human (50 g/day). The control group was treated with the solvent. Before treatment, the average body weight of mice was regarded as 16 g. All mice underwent gavage administration for 7 days and the detailed clinical sighs of toxicity, including mortality, changes in body weight skin and fur, psyche states and behavior patterns were monitored and recorded during experiment.

### *In vivo* Antifungal Efficacy Assay

Systemic candidiasis infection caused by *C. albicans* (SC5314) was established in murine model according to a previous report (Gong et al., 2019). Cells of SC5314 were cultured in liquid YPD medium plates at 30°C for 12 h. Cells were collected and washed twice with PBS, and then were adjusted to 1 × 10^7^ cells/ml in PBS. Mice were randomly divided into control group, model group and CHGX group. There were 10 mice in each group. Mice in model group and CHGX group were intravenously injected with 2 × 10^6^ cells of SC5314 in 200 μl PBS. After infection, mice in CHGX group were orally administered with 6.5 g/kg/day CHGX water-decoction for 7 days. Mice in control group and model group were treated with the solvent. The mortality of mice was monitored and recorded every 12 h during experiment.

### Statistical Analysis

Data were presented as mean ± SD. The statistical software SigmaStat package (SPSS26.0 Inc.) was used for data analysis. One-way analysis of variance (ANOVA) was performed to compare the statistical differences of data among three or more groups. *p < 0.05 was considered statistically significant.

## Results

### Identification of Major Compounds of CHGX

**FIGURE 1:**
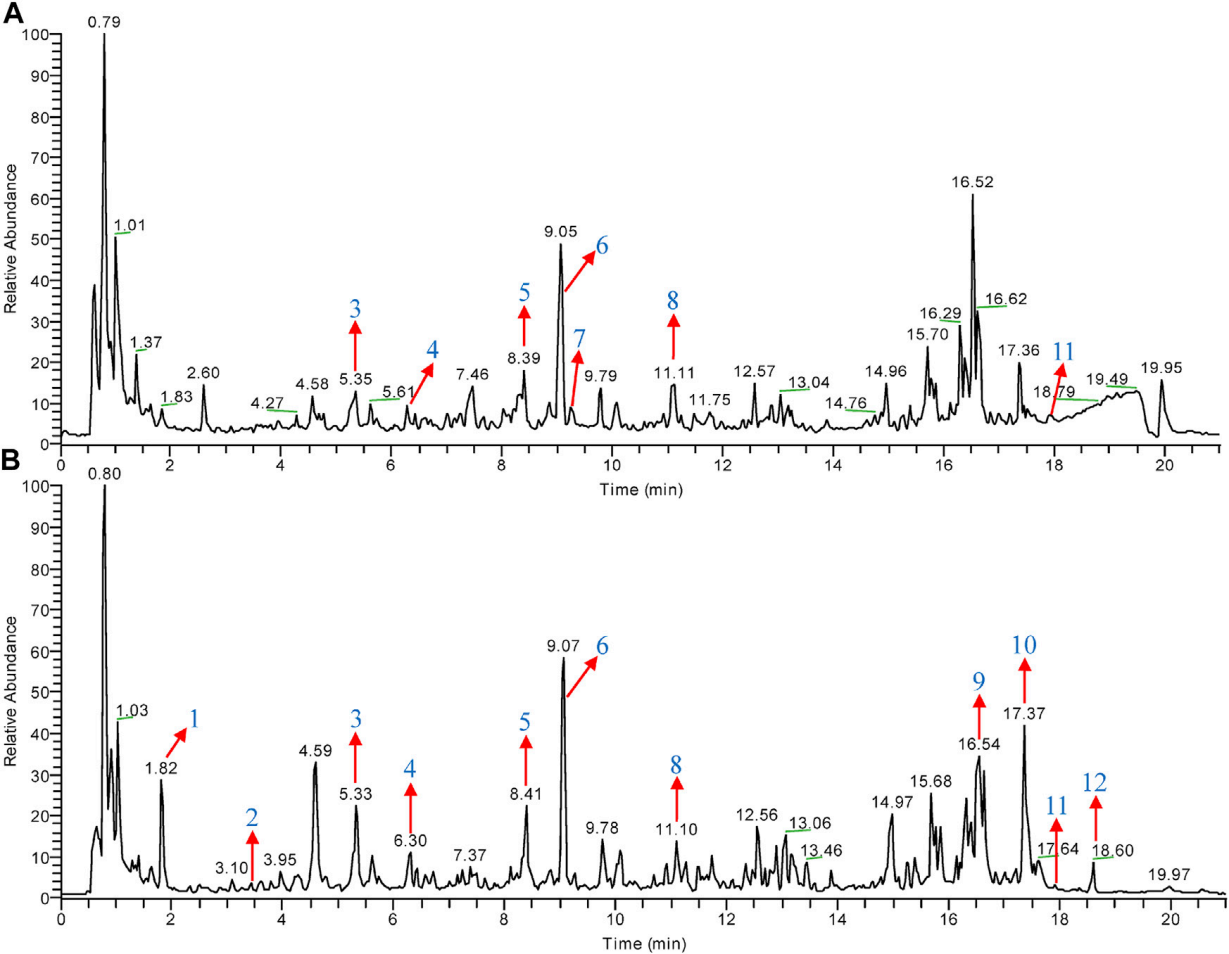
Total ion chromatogram (TIC) of CHGX analyzed by UPLC-MS/MS. UPLC-MS/MS was used for the analysis of CHGX, and data were proceeded by Xcalibur 4.2 software. The positive ion **(A)** and negative ion **(B)** chromatograms of CHGX are shown as indicated. Major compounds of CHGX were as follows: **1**, Gallic acid; **2**, Protocatechuic acid; **3**, Catechin; **4**, Epicatechin; **5**, Liquiritin; **6**, Isoliquiritin; **7**, Coumarin; **8**, Ononin; **9**, Glycyrrhizic acid; **10**, Rheic acid; **11**, Pogostone; **12**, Emodin.

CHGX water-extract was obtained from CHGX water-decoction with a yield of 16% (m/v), and it was analyzed by UPLC-MS/MS to investigate the major ingredients of CHGX. As shown in Figure 1, the total positive ion and negative ion chromatograms of samples illustrated the composition of ingredients in the CHGX. Among them, 12 major compounds, including gallic acid, protocatechuic acid, catechin, epicatechin, liquiritin, isoliquiritin, coumarin, ononin, glycyrrhizic acid, rheic acid, pogostone, and emodin were identified and characterized in Supplementary Table S1.

### Fungicidal Activity of CHGX Against *C. albicans*

**FIGURE 2:**
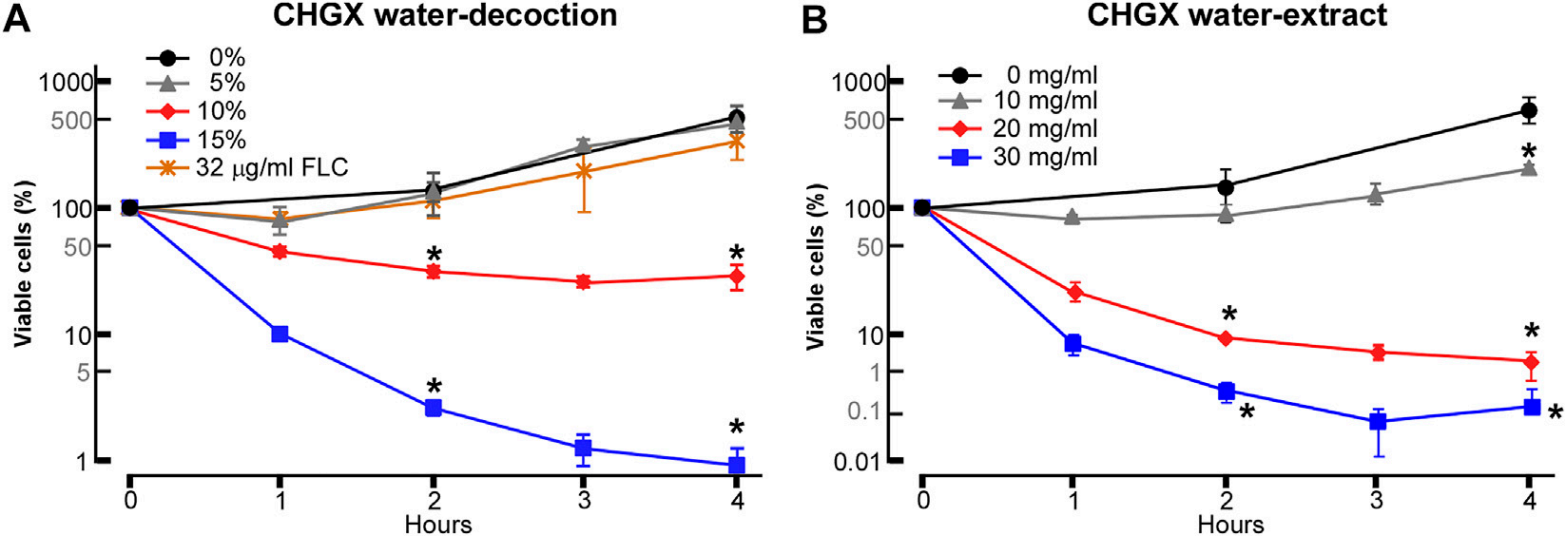
Killing activity of CHGX against *C. albicans*. SC5314 cells were used for representative strain of *C. albicans*. SC5314 cells were initially cultured in liquid YPD medium at logarithmic phase, and were then harvested, washed, and re-suspended in liquid Lee’s glucose medium for time-kill kinetics assay. SC5314 cells (2 × 10^5^ cells/ml) were treated with various concentrations of CHGX water-decoction **(A)** or CHGX water-extract **(B)** for 0–4 h at 30°C in Lee’s glucose medium, and the percentages of viable cells were determined using plating assays. Fluconazole (FLC) was used as control. Three biological repeats were performed, and the values are presented as mean ± SD. One-way analysis of variance (ANOVA) was used to compare differences between CHGX water-decoction or CHGX water-extract untreated samples and CHGX water-decoction or CHGX water-extract treated samples; *, *p* < 0.05.

The antifungal activities of CHGX water-decoction and CHGX water-extract against *C. albicans* were determined by testing of MIC. As presented in Table 1, the MIC values of CHGX water-decoction and CHGX water-extract were 12.8% (v/v) and 20 mg/ml against *C. albicans*, respectively. The MIC of AMPB was 0.25 μg/ml as a positive control. The time-kill kinetics assay showed that the CHGX exhibited noticeable fungicidal activity against *C. albicans* in a time- and dose-dependent manner as presented in Figures 2A,B. At 10% and 15% (v/v), approximately 70% and 98% of *C. albicans* cells were killed after 2 h of treatment with CHGX water-decoction in Lee’s glucose medium, respectively. Similarly, at 20 and 30 mg/ml, about 92% and 99% of *C. albicans* cells were killed after 2 h of treatment with CHGX water-extract in Lee’s glucose medium, respectively. Besides, the low dose of CHGX water-extract (10 mg/ml) also showed fungistatic activity against *C. albicans* compared with that of CHGX-untreated group.

**TABLE 1:** Antifungal susceptibility testing of CHGX formula against *C. albicans*.

	CHGX Water-decoction (%)	CHGX Water-extract	AMP B
MIC	12.8	20 mg/ml	0.25 μg/ml

Temperature and nutrition are two key environmental factors, affecting the growth of *C. albicans*. We further examined the antifungal activity of CHGX water-extract against *C. albicans* at an optimum temperature of 37°C and in a nutrient-enriched YPD medium. As shown in Supplementary Figures S1A,B, CHGX water-extract exhibited remarkable fungicidal activity both at the optimum temperature and in the nutrient-enriched medium, suggesting that CHGX possessed a stable antifungal activity in respond to environmental fluctuation.

Morphological diversity is a typical biological feature of *C. albicans* that enhances its growth in several cellular forms, including the unicellular budding yeast and filamentous hyphal cells, so as to adapt to various circumstances (Biswas et al., 2007; Sudbery, 2011; Gow et al., 2012; Huang, 2012). As mentioned above, the remarkable antifungal activity of CHGX was mainly focused on the yeast cells of *C. albicans*. Thus, we further explored the antifungal activity of CHGX water-extract against hyphal cells of *C. albicans*. As expected, we found that the CHGX water-extract also exhibited a great killing activity against hyphal cells of *C. albicans* (Supplementary Figure S1C).

### Antifungal Activity of CHGX Against Azole-Resistant *C. albicans* Strains and Other Clinical *Candida* Species

**FIGURE 3:**
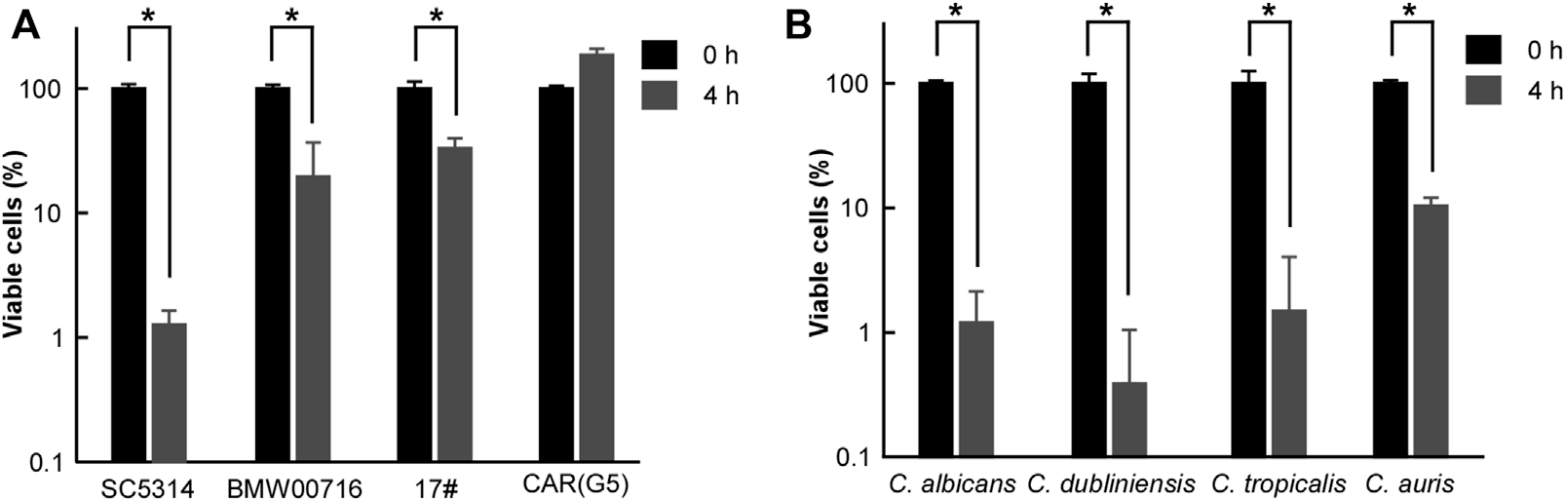
Antifungal effects of CHGX water-extract on azole-resistant strains of *C. albicans*, and other clinical fungal species. Cells of azole-resistant strains **(A)** or clinical strains **(B)** of *C. albicans* (P37005), *C. dubliniensis* (D173), *C. tropicalis* (JX1002), *C. auris* (BJCA001) were initially cultured in liquid YPD medium at logarithmic phase, and were then harvested, washed, and re-suspended in liquid Lee’s glucose medium for a time-kill kinetics assay. Fungal cells (2 × 10^5^ cells/ml) were treated with 20 mg/ml GHGX water-extract for 0 h and 4 h at 30°C in Lee’s glucose medium, and the percentage of viable cells was determined using plating assays. Three biological repeats were performed, and the values are presented as mean ± SD. One-way analysis of variance (ANOVA) was employed to compare differences between 0 and 4 h as indicated; *, *p* < 0.05.

Using a similar strategy, we investigated the antifungal activity of CHGX against azole-resistant *C. albicans* strains [BMW00716, 17#, and CAR (G5)]. Surprisingly, at 20 mg/ml, approximately 70% of cells of BMW00716 and 17# strains were killed after 4 h of treatment with GHGX water-extract (Figure 3A). Moreover, although CHGX water-extract didn’t have fungicidal effect, but still showed fungistatic effect on the growth of CAR (G5) strain (data not shown). These results indicated that CHGX possessed a potent antifungal effect against azole-resistant *C. albicans* strains.

Furthermore, we tested the killing effect of CHGX water-extract against other clinical fungal species, including *Candida dubliniensis*, *Candida tropicalis*, and *Candida auris*. As illustrated in Figure 3B, at 20 mg/ml, about 90% of fungal cells of *C. dubliniensis*, *C. tropicalis*, and *C. auris* were killed in a manner similar to that of *C. albicans* after 4 h of treatment with CHGX water-extract, suggesting that CHGX had a broad-spectrum fungicidal activity against various *Candida* species.

### Inhibitory Effects of CHGX on Biofilm Formation and Filamentous Growth of *C. albicans*

**FIGURE 4:**
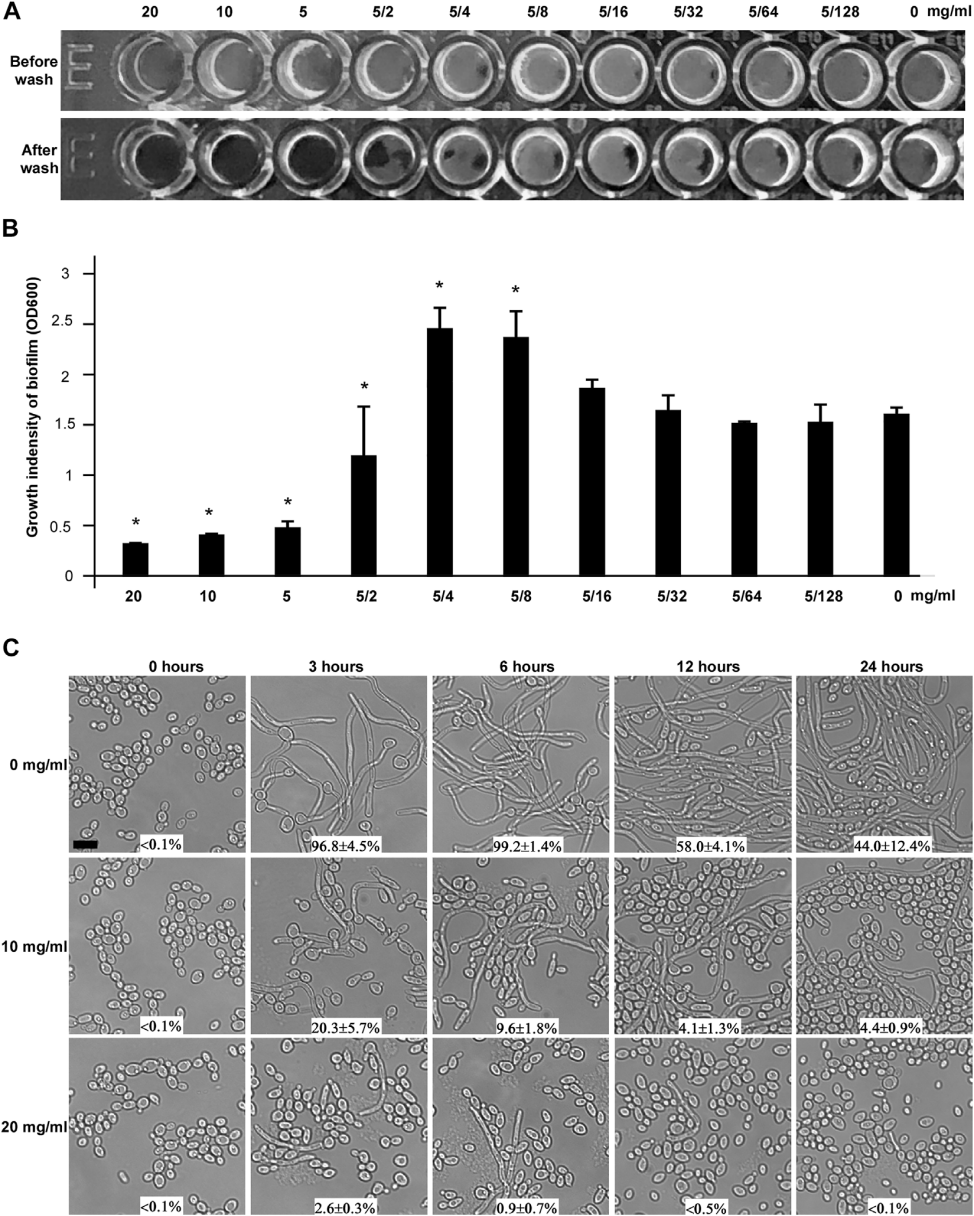
The effects of CHGX on biofilm formation and yeast-to-hyphal transition by *C. albicans*. **(A)** Biofilm formation. The minimal inhibitory concentration of CHGX water-extract for biofilm formation was determined in a flat-bottom 96-well plate by the microdilution method. SC5314 cells were used for representative strain of *C. albicans.* 1 × 10^5^ cells of SC5314 were incubated with different concentrations of CHGX water-extract (20–0.04 mg/ml) in a 200 μl Spider medium per well for 3 days with shaking at 37°C. The plates were imaged before and after being gently washed with 1 × PBS, and three biological repeats were performed. **(B)** The growth intensity of biofilm. Cells adhered to the bottoms of 96-well plates were treated with 10% trypsin, washed, collected and resuspended in total 600 μl PBS. The cells density (OD600) was read with Synergy 4 Gene 5 plate reader for quantitation of biofilm. One-way analysis of variance (ANOVA) was used to compare differences between CHGX water-extract treated and untreated samples (control) as indicated; *, *p* < 0.05. **(C)** Yeast-to-hyphal transition. Yeast cells of *C. albicans* (SC5314, 2 × 10^6^ cells/ml) were treated with 0, 10 or 20 mg/ml CHGX water-extract at 37°C in Lee’s glucose medium supplemented with 10% FBS. The cellular morphology and the percentage of hyphal cells were determined by microscopic observation at 0, 3, 6, 12 and 24 h. Three biological repeats were performed (scale bar, 10 μm).

The ability of *C. albicans* to form biofilm is considered as a crucial virulence factor in terms of its contribution to human diseases (Fanning and Mitchell, 2012; Sardi et al., 2013). The study of biofilm formation is vital to explore antifungal properties of natural products. Hence, we attempted to test whether CHGX could affect the biofilm formation ability of *C. albicans*. As shown in Figure 4A, *C. albicans* could not form a mature biofilm on the plastic bottom in the presence of 20, 10, and 5 mg/ml of CHGX water-extract. The growth intensity of biofilm in the presence of different concentration of CHGX was also indicated in Figure 4B. As mentioned above, the MIC value of CHGX water-extract was 20 mg/ml against *C. albicans* which was much higher than the minimum inhibitory concentration of biofilm (5 mg/ml). Therefore, the inhibitory effect of biofilm formation was due to the anti-biofilm activity, rather than the growth inhibitory activity of CHGX.

The ability to switch between yeast and filamentous hyphal cells not only is tightly associated with biofilm formation, but also plays a significant role in virulence and pathogenesis of *C. albicans* (Sudbery, 2011; Noble et al., 2017). To explore the effects of CHGX on morphogenesis, yeast-to-filamentous transition was examined. Serum is a robust factor inducing filamentation in *C. albicans* (Hudson et al., 2004). After incubation for 3 h in Lee’s glucose medium supplemented with 10% fetal bovine serum (FBS), *C. albicans* yeast cells almost completely (>90%) converted to hyphal cells in the absence of CHGX water-extract, whereas a remarkably smaller percentage of hyphal cells were observed in the presence of CHGX water-extract in a dose-dependent manner, and the percentage of hyphal cells gradually decreased with the extension of incubation time (Figure 4C). The inhibitory effect of yeast-to-filamentous transition was also confirmed in RMMI 1640 medium (Supplementary Figure S2). After incubation of 24 h in RPMI 1640 medium, the percentage of hyphal cells observed in CHGX-treated cells was significantly lower than that of CHGX-untreated cells. Taken together, these results indicated that CHGX could effectively inhibit hyphal formation, and exerted an anti-biofilm effect against *C. albicans*.

### The Effects of CHGX on the Cell Membrane Integrity and Permeability of *C. albicans*

**FIGURE 5:**
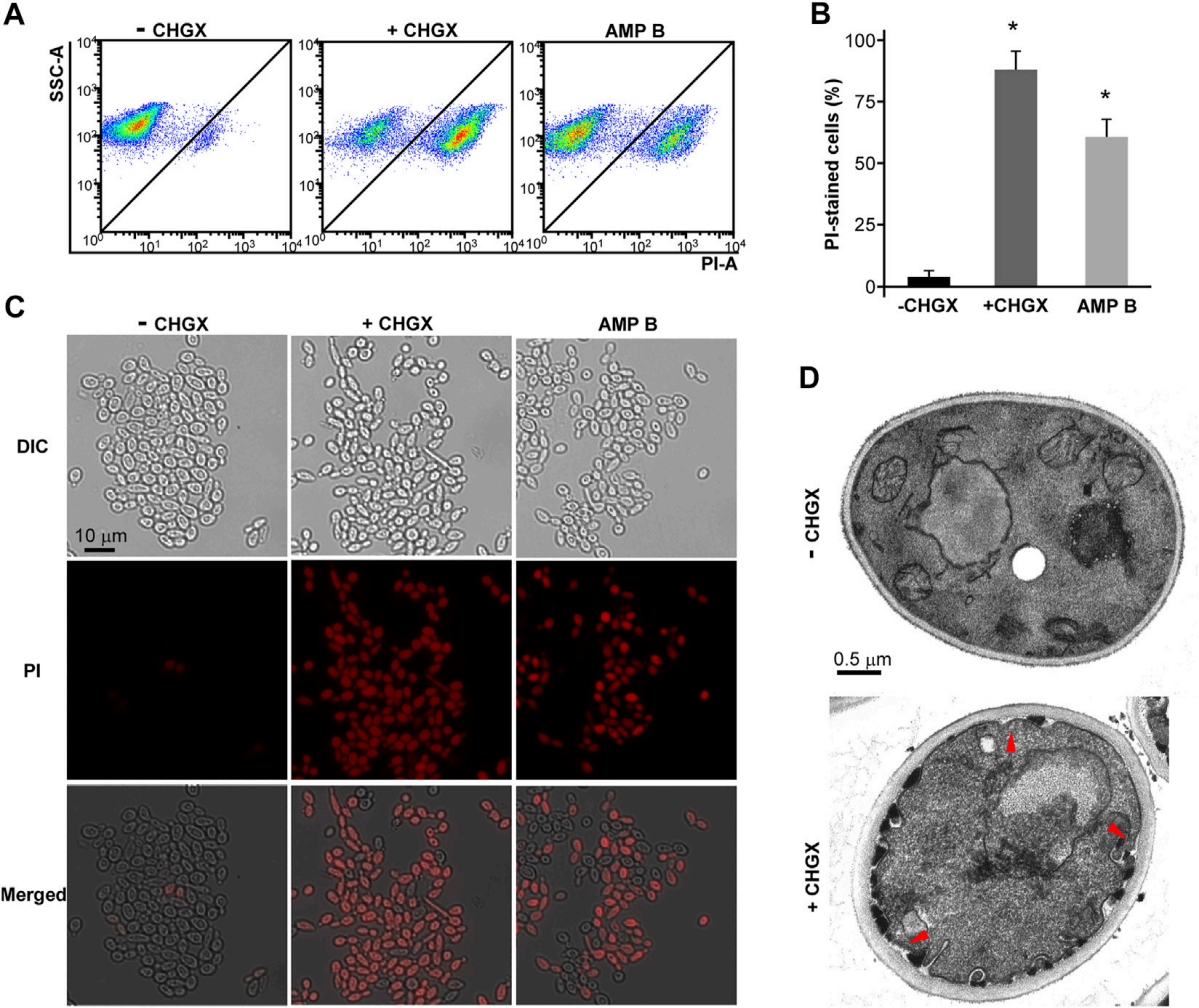
CHGX water-extract causes cell membrane damage. SC5314 cells were used for representative strain of *C. albicans.* SC5314 cells (2 × 10^6^ cells/ml) were incubated with or without 20 mg/ml CHGX water-extract in Lee’s glucose medium for 3 h at 30°C. **(A–C)** PI staining assay. Cells were harvested and stained with red PI dye for fluorescence-activated cell sorting analysis **(A)** and fluorescence microscopy **(C)**. **(B)** The percentages of PI-stained cells. AMPB-treated cells were severed as positive control. The data were presented as mean ± SD of three independent experiments according to fluorescence microscopy assay in panel C. SSC-A, side scatter area; PI-A, propidium iodide area. One-way analysis of variance (ANOVA) was used to compare differences between drug treated and untreated samples as indicated; *, *p* < 0.05. **(D)** TEM assay. The red arrows indicated cellular membrane damage.

To explore the antifungal mechanism of CHGX, flow cytometry and microscopic analysis were conducted to examine the effects of CHGX on the cell membrane integrity. PI dye is unable to cross cell membranes, while it can enter damaged membranes and bind to DNA to produce red fluorescence (Du et al., 2015). Firstly, we treated cells with or without 20 mg/ml of CHGX water-extract for 3 h in Lee’s glucose medium. We also treated cells with 2 μg/ml AMPB for 1 h, as a potent fungicidal agent. Flow cytometry assay demonstrated that the treatment of CHGX water-extract led to the uptake of dye PI in *C. albicans* (Figure 5A). These results were further confirmed by fluorescence microscopy (Figures 5B,C), displaying that the PI uptake of CHGX water-extract treated cells and CHGX water-extract untreated cells were about 87 and 3%, respectively. The PI uptake of AMPB-treated cells was approximately 60%. These results suggested that CHGX could lead to the impairment of membrane integrity.

To validate this hypothesis, we performed transmission electron microscopy (TEM) assay. TEM assay revealed a normal morphology in untreated cells, while a large number of thick-walled cells, as well as invagination and disintegration of plasma membrane were observed after treatment with CHGX water-extract for 3 h (Figure 5D). The disruption of membrane integrity may lead to the increased cell permeability and cause cell leakage of intracellular components, ultimately resulting in cell death of *C. albicans* under the treatment of CHGX.

### The Effects of CHGX on the Intracellular ROS Production and ATP Level

**FIGURE 6:**
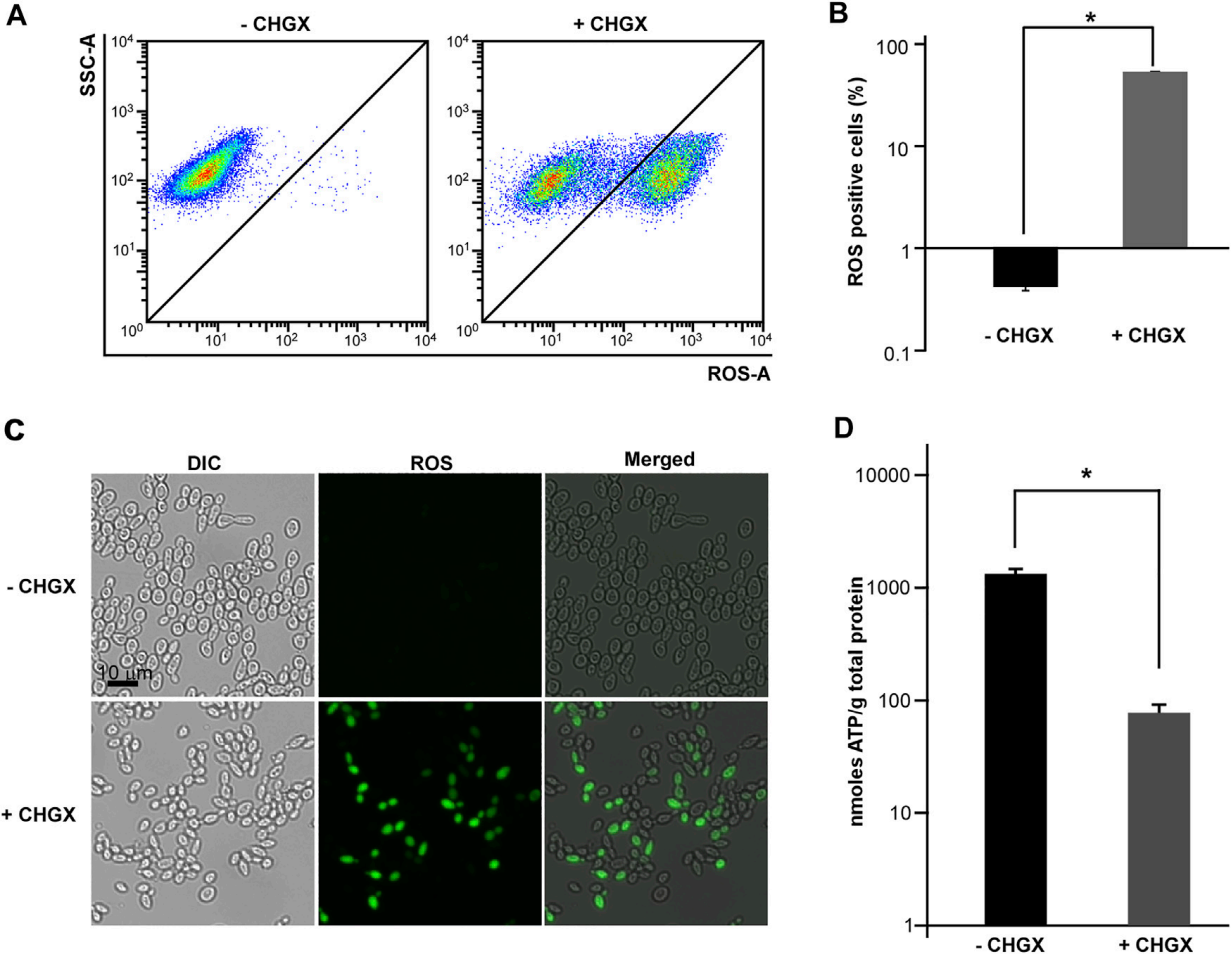
The effects of CHGX water-extract on intracellular ROS production and ATP consumption by *C. albicans*. SC5314 cells were used for representative strain of *C. albicans.* SC5314 cells (2 × 10^6^ cells/ml) were treated with or without 20 mg/ml CHGX water-extract in Lee’s glucose medium for 3 h at 30°C. **(A–C)** The intracellular accumulation of ROS. Cells were harvested and incubated with DCFH-DA for flow cytometry **(A)** and fluorescence microscopy **(C)**. SSC-A, side scatter area; ROS-A, ROS area. **(B)** The percentages of ROS-positive cells. The data were presented as mean ± SD of three independent experiments according to flow cytometry assay in panel A. **(D)** The level of intracellular ATP. Cells were harvested and homogenized in 1 × PBS for ATP assay by an ATP Bioluminescence Assay kit. Three biological repeats were performed, and the values are presented as mean ± SD. One-way analysis of variance (ANOVA) was used to compare differences between CHGX water-extract treated and untreated samples as indicated; *, *p* < 0.05.

The ROS production is a common mechanism of antifungal medications. Given the important role of ROS production in antifungal agents, we supposed that ROS production was critical for CHGX-induced cell death in *C. albicans*. We measured the production of ROS using a DCFH-DA detection kit in *C. albicans*, which could be oxidized by intracellular radicals to excite the fluorescence signals. After 3 h of treatment, the results of flow cytometry showed that CHGX water-extract significantly induced ROS-fluorescence signals compared with the control group (Figures 6A,B). These results were additionally confirmed by fluorescence microscopy (Figure 6C), indicating that the production of ROS contributed to the fungicidal activity of CHGX against *C. albicans*.

Cellular ATP levels can change dramatically in response to sever stress conditions, including antifungal drugs which trigger fungal cell death (Osório et al., 2004; Kohanski et al., 2007; Belenky et al., 2013). Next, we examined the intracellular ATP levels under the treatment with 20 mg/ml CHGX water-extract. As demonstrated in Figure 6D, the level of intracellular ATP in CHGX water-extract treated cells was extremely lower than that of untreated cells, implying that CHGX may involve metabolic changes, and decreased ATP production or increased ATP consumption in *C. albicans*.

### The Regulatory Role of Ras1-cAMP Pathway in CHGX-Induced Cell Death

**FIGURE 7:**
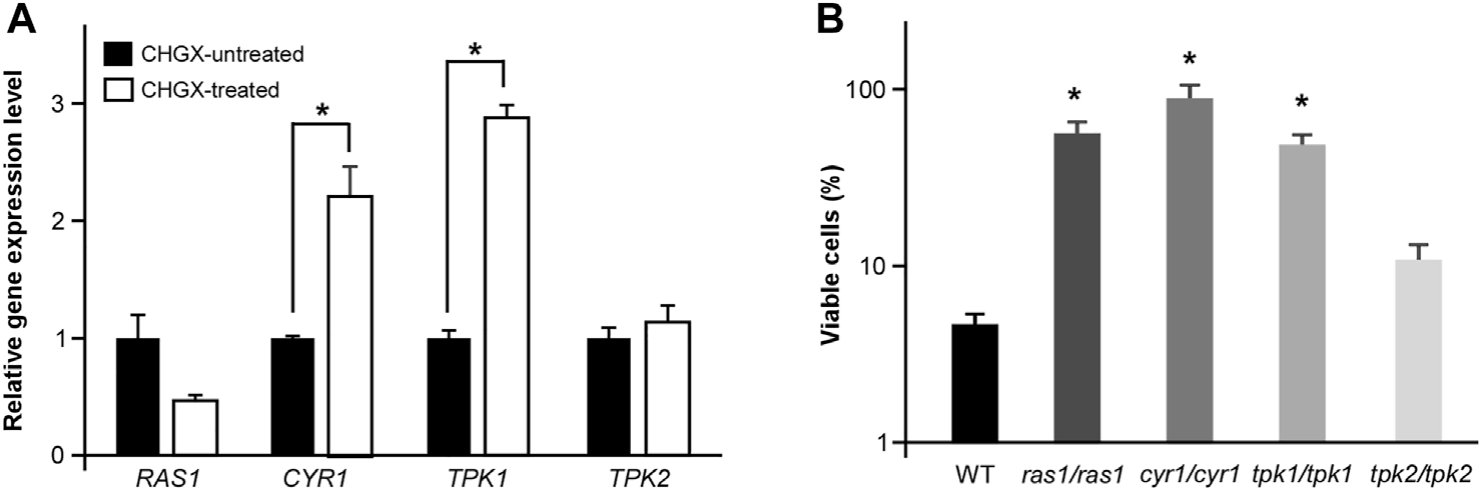
The cAMP-PKA signaling pathway regulates CHGX water-extract-induced cell death of *C. albicnas*. **(A)** Relative expression levels of *RAS1*, *CRY1*, *TPK1,* and *TPK2* in CHGX water-extract treated and untreated SC5314 cells. Total RNA was extracted from cells grown in Lee’s glucose medium with or without CHGX water-extract (20 mg/ml) at 30°C for 3 h and used for qRT-PCR assay. The relative gene expression level in CHGX water-extract-untreated cells was set as “1”. **(B)** The cell viability assay. Strains used were the WT (SN152), *ras1/ras1*, *cyr1/cyr1*, *tpk1/tpk1*, *tpk2/tpk2* mutants. Fungal cells (2 × 10^5^ cells/ml) were treated with 20 mg/ml CHGX water-extract in Lee’s glucose medium for 3 h at 30°C. The percentage of viable cells was determined using plating assays. Three biological repeats were performed, and the values are presented as mean ± SD. One-way analysis of variance (ANOVA) was used to compare differences between WT and mutant strains; *, *p* < 0.05.

**FIGURE 8:**
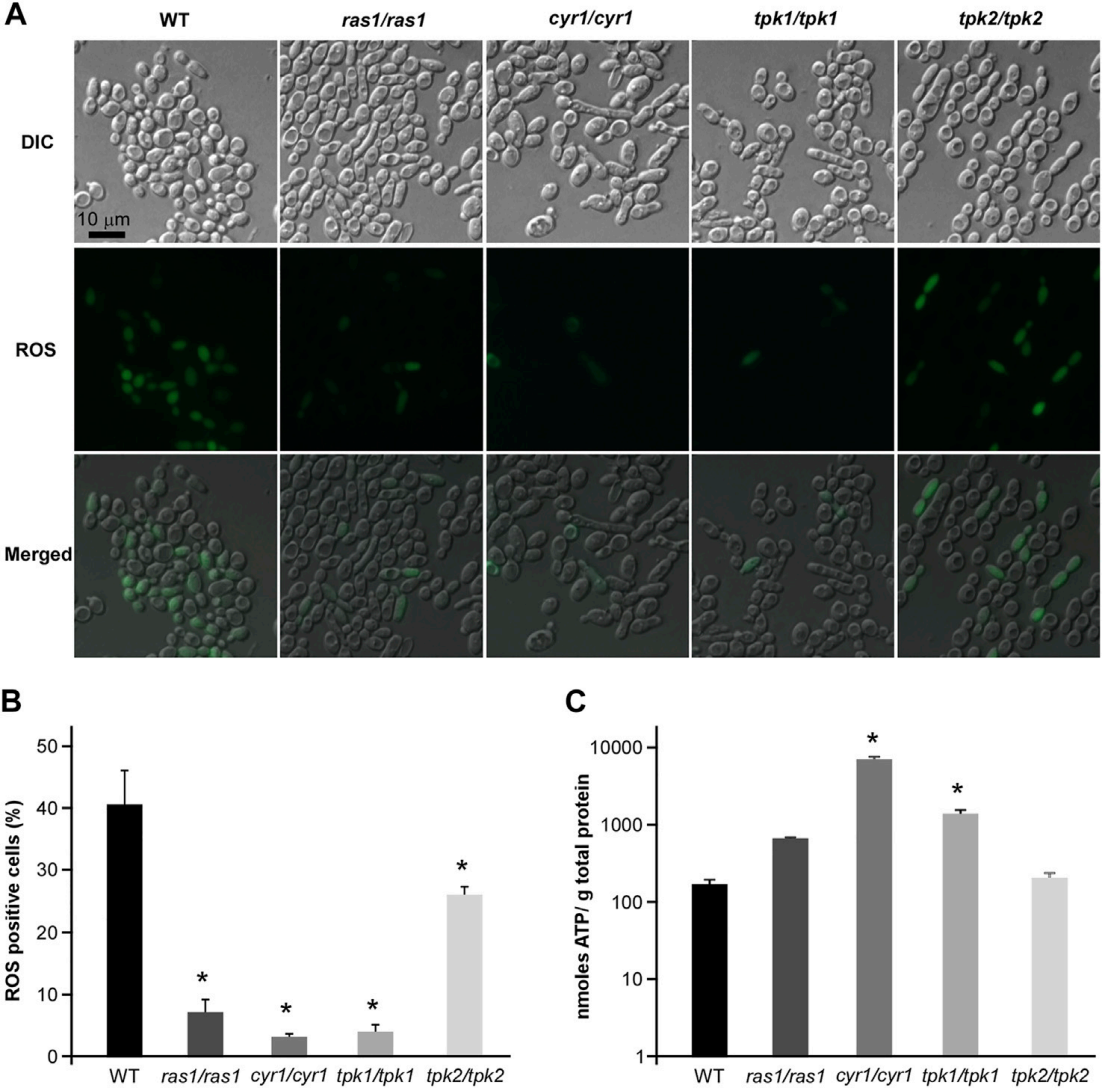
The regulatory role of cAMP-PKA pathway in ROS accumulation and ATP consumption in CHGX water-extract-induced cell death of *C. albicnas*. Strains used were the WT (SN152), and *ras1/ras1*, *cyr1/cyr1*, *tpk1/tpk1*, *tpk2/tpk2*, *tpk1/tpk1 tpk2/tpk2* mutants. **(A)** The intracellular ROS production in WT and mutant strains. Fungal cells (2 × 10^6^ cells/ml) were treated with 20 mg/ml CHGX water-extract in Lee’s glucose medium for 3 h at 30°C. Cells were harvested and incubated with DCFH-DA for fluorescence microscopy. **(B)** The percentage of ROS-positive cells. The values were expressed as mean ± SD of three independent experiments according to fluorescence microscopy in panel A. **(C)** The intracellular ATP level in WT strain and mutants. Fungal cells (2 × 10^6^ cells/ml) were treated with 20 mg/ml GHGX water-extract in Lee’s glucose medium for 2 h at 30°C. Cells were harvested and homogenized with 1× PBS for ATP assay by an ATP Bioluminescence Assay kit. Three biological repeats were performed, and the values are presented as mean ± SD. One-way analysis of variance (ANOVA) was used to compare differences between WT and mutant strains as indicated in panels B and C; *, *p* < 0.05.

The conserved Ras1-cAMP pathway plays a key role in the regulation of several biological aspects, involving in cell growth and death, pathogenesis, morphological transition and stress responds in *C. albicnas* (Huang et al., 2019). Therefore, we attempted to explore the regulatory role of Ras1-cAMP pathway in CHGX-induced cell death in *C. albicans*. Firstly, We observed that the relative expression levels of *CYR1* and *TPK1* in Ras1-cAMP pathway were significantly up-regulated after CHGX water-extract treatment (Figure 7A). We further examined the role of the Ras1-cAMP pathway in CHGX-induced cell death by studying single-gene knockout. As expected, the susceptibility of the cells of the *ras1/ras1*, *cyr1/cyr1* and *tpk1/tpk1* mutants to CHGX water-extract significantly decreased compared with that of the wild-type (WT) strain according to the killing assay (Figure 7B). Furthermore, we found that the CHGX-dependent ROS accumulation was markedly reduced in the cells of the mutants via the Ras1-cAMP pathway (Figures 8A,B). Consistently, the intracellular ATP level was remarkably elevated in the cells of *cyr1/cyr1* and *tpk1/tpk1* mutants after CHGX water-extract treatment (Figure 8C). Taken together, these results indicated that the Ras1-cAMP pathway played a critical role in CHGX-induced cell death of *C. albicans* by mediating the mitochondrial ROS production and ATP consumption.

### The Mitochondrial Protein Mcu1 Could Regulate CHGX-Induced Cell Death

**FIGURE 9:**
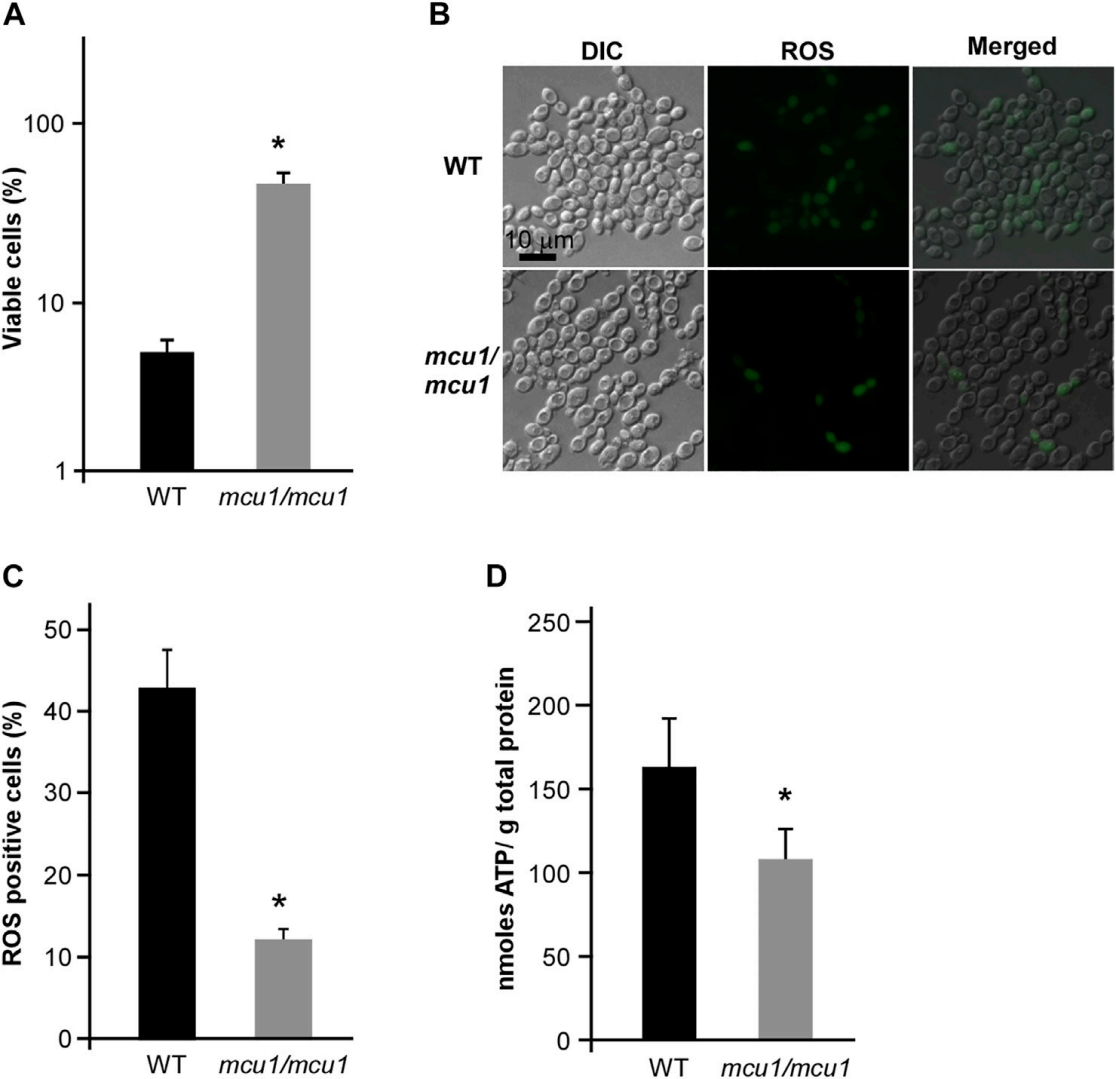
The mitochondrial protein Mcu1 regulates CHGX water-extract-induced cell death of *C. albicans*. **(A)** The cell viability assay of WT and *mcu1/mcu1* mutant strains. Fungal cells (2 × 10^5^ cells/ml) were treated with 20 mg/ml CHGX water-extract in Lee’s glucose medium for 3 h at 30°C. The percentage of viable cells was determined using plating assays. **(B)** The intracellular ROS production in WT and *mcu1/mcu1* mutant strains. Fungal cells (2 × 10^6^ cells/ml) were treated with 20 mg/ml CHGX water-extract in Lee’s glucose medium for 3 h at 30°C. Cells were harvested and incubated with DCFH-DA for fluorescence microscopy. **(C)** The percentage of ROS-positive cells. The values were expressed as mean ± SD of three independent experiments according fluorescence microscopy assay in panel B. **(D)** The intracellular ATP level of WT and *mcu1/mcu1* mutant strains. Fungal cells (2 × 10^6^ cells/ml) were treated with 20 mg/ml CHGX water-extract in Lee’s glucose medium for 2 h at 30°C. Cells were harvested and homogenized with 1× PBS for ATP assay by an ATP Bioluminescence Assay kit. Three biological repeats were performed, and the values are presented as mean ± SD. One-way analysis of variance (ANOVA) was used to compare differences between WT and *mcu1/mcu1* mutant strains; *, *p* < 0.05.

Mcu1 is an important mitochondrial protein involved in carbon source utilization, filamentation, and virulence in *C. albicans* (Du et al., 2015; Guan et al., 2015). In the present study, we found that deletion of *MCU1* in *C. albicans* significantly increased cell viability after treatment with CHGX water-extract (Figure 9A). Additionally, the intracellular ROS production and ATP level in cells of *muc1/mcu1* mutant exhibited corresponding response patterns to the CHGX water-extract treatment according to the results of cell viability assay (Figures 9B–D). These findings confirmed the key role of Mcu1 in the regulation of CHGX-induced cell death in *C. albicans*.

### *In vivo* Toxicity Evaluation and Antifungal Efficacy of CHGX

**FIGURE 10:**
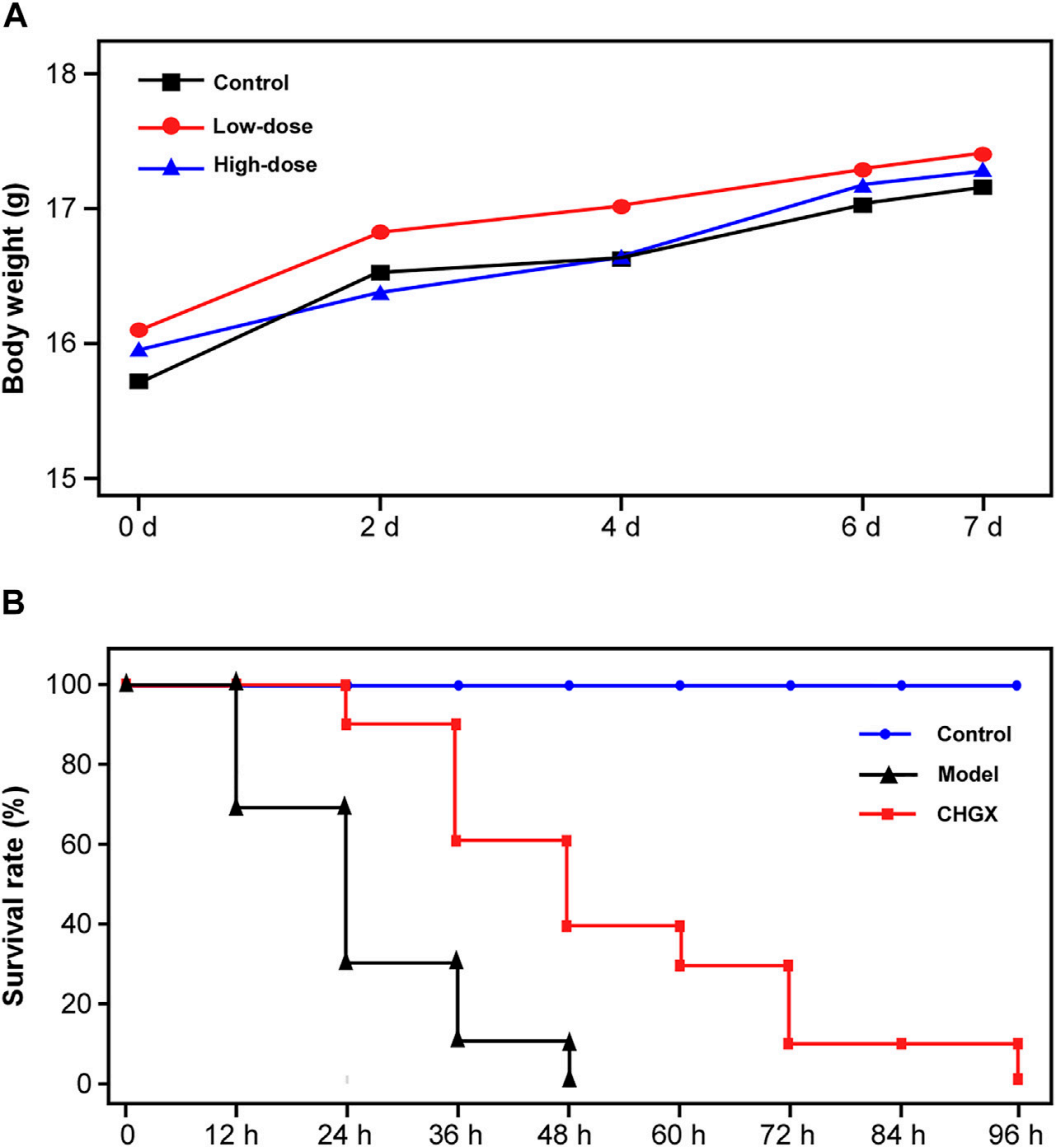
The *in vivo* toxicity assay and antifungal efficacy of CHGX in murine model. **(A)** Effect of different dose of CHGX on the body weight in mice. Mice were randomly divided into control groups, low-dose (6.5 g/kg/day) group and high-dose (13 g/kg/day) group, five mice were used in each group. During the 7 days of treatment, the body weight of each mouse was recorded, and the average body weight was calculated and presented. **(B)** Kaplan–Meier curves for the survival rate of candidiasis mice. Mice in model and CHGX groups were intravenously injected with 2 × 10^6^ cells of SC5314 in 200 μl PBS. The CHGX group were orally administered with 6.5 g/kg/day CHGX water-decoction for 7 days, and the control and model groups were treated with the solvent. The mortality of mice was monitored and recorded every 12 h during experiment.

Initially, the murine model was used to evaluate the toxicity of CHGX water-decoction at doses of 6.5 g/kg and 13 g/kg. We found that there was no mortality and noticeable clinical signs of toxic effects on mice at the tested concentrations throughout the 7 days. All mice behaved normally without altered psyche states and body appearance, suggesting that the CHGX treatment did not induce any significant morphological changes. The body weight is a critical indicator of the adverse effects of drugs. Before treatment, the body weight was 15.22–16.71 g in mice. During the 7 days of experiment period, the body weight gradually increased in all groups and there was no significant difference between the control group and experimental groups (Figure 10A).

The *in vivo* antifungal effect of CHGX on systemic candidiasis murine model was shown in Figure 10B. In the model group, all mice were died within 48 h. When the mice were administrated with CHGX water-decoction (6.5 g/kg) after *C. albicans* infection, the survival rate of mice was significantly improved. These results indicated that CHGX had *in vivo* efficacy on treating *C. albicans* infection. However, all mice in the CHGX group died within 96 h, due to the severity of the systemic candidiasis infection in murine. Hence, further animal experiments and clinical trials are need to validate the *in vivo* efficacy of CHGX.

## Discussion

Medicinal plants are important sources for the treatment of severe pathogenic infections, which can be associated with the presence of bioactive compounds in TCM. The emergence of resistant fungal pathogens and the limited choice of antifungal drugs motivate scholars to explore and develop alternative antifungal agents. As a major human fungal pathogen, *C. abicans* has become one of the key risk factors of death in patients with septic shock related to *Candida* infections, especially in patients who are hospitalized in intensive care units (ICUs) (Pfaller and Diekema, 2010; Brown et al., 2012; Kullberg and Arendrup, 2015). CHGX is an empirical prescription for the treatment of *Candida* infections, which has been clinically applied for more than a decade. The CHGX was designed and evolved based on Qingquan Liu’s research, as the leading research for the therapy of septic patients using TCM (Zhao et al., 2019). However, there is a lack of a large and high-quality randomized controlled trial for the efficacy evaluation of CHGX in patients with *Candida*-related infections. To investigate whether the beneficial therapeutic effects of CHGX can be attributed to the antifungal activity of CHGX, we, for the first time, studied the anti-*Candida* properties of CHGX, and found its antifungal mechanisms against *C. albicans*. In this study, we demonstrated that the CHGX exhibited a fungicidal activity against *C. albicans* and other clinical *Candida* species. The antifungal activity was even observed against fluconazole-resistant *C. albicans* strains. Besides, the *in vivo* toxicity showed that CHGX could be used safely without distinct toxicity or side effects. Combined with previous clinical findings, our study validate that CHGX formula can contribute as an alternative treatment, targeting both at solving the problems of fluconazole resistance, as well as overcoming the limitations of conventional antifungals.

Except for the direct fungicidal activity of CHGX against the cell growth of *C. albicans*, anti-biofilm activity is also an important property of antifungal drugs. Fungal biofilm is a highly complicated and constructed community where fungal cells are embedded in self-produced extracellular matrix, restricting the penetration of antifungal agents and promoting the development of drug resistance. Biofilm can grow on any biotic or abiotic surfaces, especially on implanted medical devices, leading to about half of all nosocomial fungal infections, as well as posing great challenges for the clinical treatments (Zarnowski et al., 2014; Mitchell et al., 2015; Dominguez et al., 2018). In the present study, we found that CHGX water-extract showed a noticeable anti-biofilm activity against *C. albicans*. Regarding the association between biofilm formation and drug resistance to antifungals, the anti-biofilm activity of CHGX would render the biofilm to be eradicated more easily in oral candidiasis or systemic *Candida* infections. Besides, hyphal transition not only regulates biofilm maturation, but also plays a crucial role in the pathogenesis of *Candida* infections, as hyphal cells exhibits greater adhesion and penetration abilities in human epithelial cells than yeast cells (Mukaremera et al., 2017). As expected, the CHGX showed striking inhibitory effects on filamentous growth of *C. albicans*, further confirming its pharmacological features as an alternative antifungal drug against *C. albicans*-related infections.

The cell wall and cellular membrane act as a barrier against the entry or exit of substances, and they also play a crucial role in the fungal pathogenesis. Besides, the cell membrane integrity and permeability are necessary for survival and host adaptation under fluctuant conditions during fungal infections (Latgé et al., 2017). The polyenic antifungal antibiotics, such as AMPB, form channels in the cell membrane, and cause extravasation of potassium, sodium, and carbohydrate substances, resulting in the cell death of fungus (Mesa-Arango et al., 2012; Cowen et al., 2014). Therefore, the effects of CHGX on the cell membrane integrity may be the primary mode of action against *C. albicans*. Flow cytometry and fluorescence microscopy of DNA-binding dye PI showed that CHGX could cause damage to cell membrane of *C. albicans*. This result was confirmed by TEM assay, in which *C. albicans* cells with the treatment of CHGX water-extract revealed obvious impairments of cell membrane. Thus, the antifungal mechanism of CHGX against *C. albicans* could be related to the cellular membrane disruption, accompanying with the increased permeability and intracellular content leakage, resulting in cells death of *C. albicans.*

Except for the primary modes of action, the oxidative-damage cell death is a common pathway in all the three classes of fungicidal drugs, that involves alterations to cellular respiration, leading to the accumulation of ROS (Kohanski et al., 2007; Belenky et al., 2013). The production of intracellular ROS can cause damage to cell membranes, proteins, and DNA, ultimately resulting in death of yeast cells (Phillips et al., 2003; Breitenbach et al., 2005; Belenky et al., 2013). In this study, we found that CHGX could induce ROS-dependent cell death regulated by Ras1-cAMP signaling pathway in *C. albicans*. The conserved Ras1-cAMP pathway, consisting of two GTPases (Ras1 and Ras2), an adenylyl cyclase (Cyr1, also named as Cdc35), and two PKA catalytic isoforms (Tpk1 and Tpk2), plays a critical role in the regulation of multiple biological characteristics, which are important for the virulence of *C. albicans*, such as cell growth and death, phenotypic switching, biofilm formation, and sexual reproduction (Huang et al., 2019). Previous studies reported that the Ras1-cAMP pathway could respond to antifungal agents by the regulation of ROS production and ATP consumption via mitochondrial dysfunction in *C. albicans* (Chevtzoff et al., 2010; Leadsham and Gourlay, 2010; Belenky et al., 2013). In the present study, we found that the Ras1-cAMP pathway was robustly induced after treatment with CHGX. The inactivation of Ras1-cAMP pathway made *C. albicans* less susceptible to CHGX treatment and reduced the killing activity by decreasing the production of excessive ROS and increasing the accumulation of ATP. Additionally, the *Candida*-specific mitochondrial protein, Mcu1, was involved in regulating the mitochondrial metabolism and the production of ROS. Here, we proposed that the cellular changes and damages initiated by interaction with CHGX could lead to the activation of Ras1-cAMP and other pathways, resulting in the activation of mitochondrial respiration combined with ROS production and ATP consumption, ultimately contributing to the cell death in *C. albicans*.

A limitation of our study is that the bioactive compounds responsible for the antifungal activity of CHGX have not been identified yet. We tried to prepare CHGX extract via maceration in four solvents: petroleum ether, dichloromethane, ethyl acetate and N-butanol in turn. The petroleum ether extract and dichloromethane extract of CHGX were two major active fractions (data not shown). A large-scale extract was prepared via maceration in petroleum ether and dichloromethane, followed by fractions on a Sephadex LH20 gel column to yield a total of 42 fractions. Four major active fractions (F16, F17, F18, F19) were identified (data not shown), and will be further separated by HPLC to get the active chromatographic peaks and compounds.

## Conclusion

This study highlights the antifungal activity of CHGX, a Chinese herbal medicine. Results demonstrated that CHGX exhibited efficient, stable and broad-spectrum fungicidal effect on *Candida* species and azole-resistant clinical strains. Moreover, CHGX was effective in inhibiting biofilm formation and filamentation of *C. albicans*, pointing out that CHGX would be less likely to develop drug resistance in clinical application, which can be considered as a potential antifungal agent in the management of disseminative candidiasis. Furthermore, our findings provided new insights to antifungal mechanisms of CHGX against *C. albicans* and validated the safety and efficacy of CHGX in murine model, confirming its clinical potential as antifungals. However, the bioactive molecules of CHGX remain to be investigated and further *in vivo Candida* infection models and clinical trials are required to comprehensively evaluate its pharmacological effect and clinical efficacy.

## Data Availability

The original contributions presented in the study are included in the article/Supplementary Material; further inquiries can be directed to the corresponding author.
