# Corrigendum: Antifungal mechanisms of a Chinese herbal medicine, Cao Huang Gui Xiang, against *Candida* species

**DOI:** 10.3389/fphar.2022.977030

**Published:** 2022-07-22

**Authors:** Huizhen Yue, Xiaolong Xu, Shasha He, Xuran Cui, Yuhong Guo, Jingxia Zhao, Bing Peng, Qingquan Liu

**Affiliations:** ^1^ Beijing Hospital of Traditional Chinese Medicine, Capital Medical University, Beijing, China; ^2^ Beijing Institute of Chinese Medicine, Beijing, China; ^3^ Beijing Key Laboratory of Basic Research with Traditional Chinese Medicine on Infectious Diseases, Beijing, China

**Keywords:** Cao Huang Gui Xiang formula, *Candida albicans*, antifungal activity, biofilm formation, ROS production, Ras1-cAMP pathway

In the published article, there was an error in Figure 3B as published. The heading for columns 3 of Figure 3B in the published article was incorrectly listed as “*C. glabrata*.” The correct heading for columns 3 of Figure 3B is “*C. tropicalis*.” The correct Figure 3 appears below.

**FIGURE 3:**
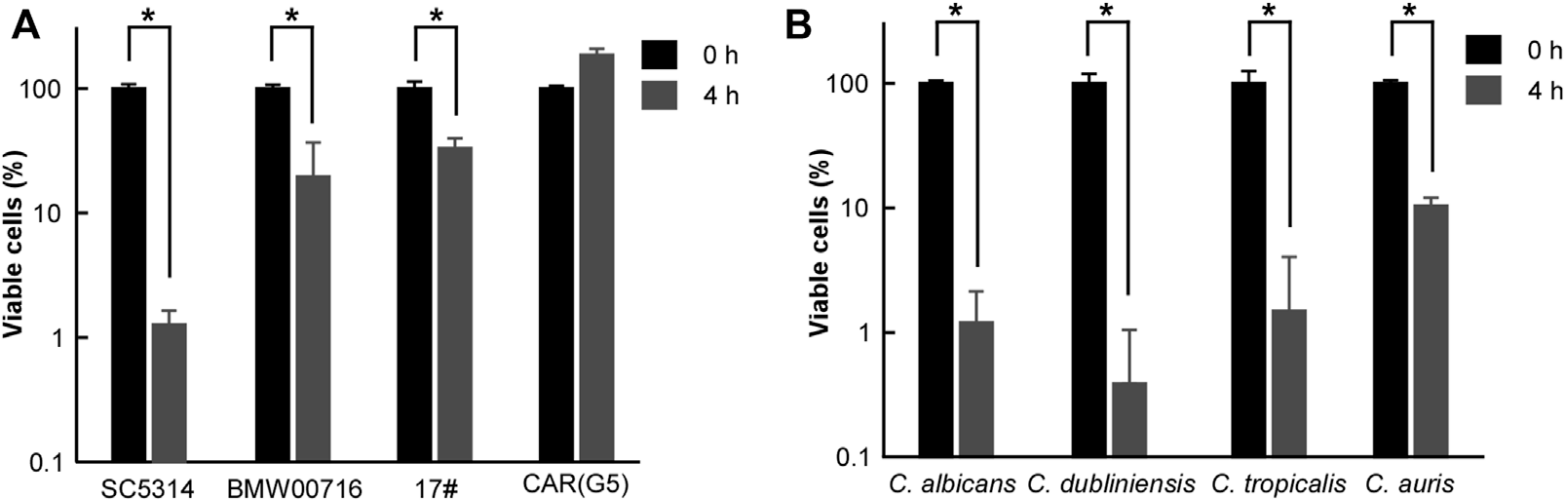
Antifungal effects of CHGX water-extract on azole-resistant strains of *C. albicans*, and other clinical fungal species. Cells of azole-resistant strains **(A)** or clinical strains **(B)** of *C. albicans* (P37005), *C. dubliniensis* (D173), *C. tropicalis* (JX1002), *C. auris* (BJCA001) were initially cultured in liquid YPD medium at logarithmic phase, and were then harvested, washed, and re-suspended in liquid Lee’s glucose medium for a time-kill kinetics assay. Fungal cells (2 × 10^5^ cells/ml) were treated with 20 mg/ml GHGX water-extract for 0 h and 4 h at 30°C in Lee’s glucose medium, and the percentage of viable cells was determined using plating assays. Three biological repeats were performed, and the values are presented as mean ± SD. One-way analysis of variance (ANOVA) was employed to compare differences between 0 and 4 h as indicated; *, *p* < 0.05.

The authors apologize for this error and state that this does not change the scientific conclusions of the article in any way. The original article has been updated.

