# Supplementary material for: Antifungal Mechanisms of a Chinese Herbal Medicine, Cao Huang Gui Xiang, Against Candida Species
Source: Front Pharmacol. 2022 Mar 9;13:813818. doi: 10.3389/fphar.2022.813818 (PMC8959912; doi:10.3389/fphar.2022.813818)
Supplement: Supplementary file 1 [file Table1.DOCX]

***Supplementary Material***

**Supplementary Figures**

**
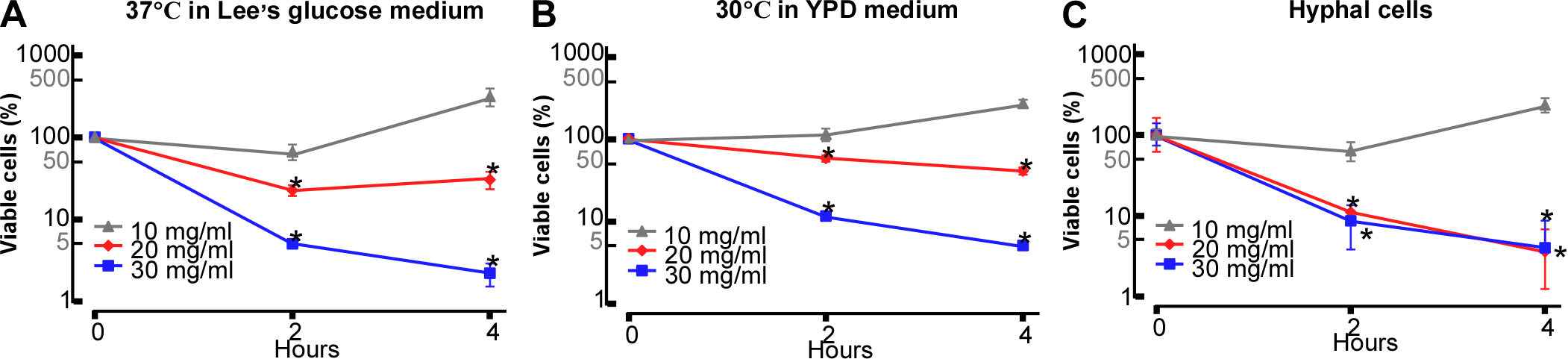
**

**Supplementary Figure 1. Antifungal activity of CHGX against *C. albicans*.** **(A-B)** Killing of *C. albicans* yeast cells by CHGX water-extract. SC5314 yeast cells were initially cultured in liquid YPD medium at a logarithmic phase, and were then harvested, washed, and re-suspended in liquid Lee’s glucose or YPD medium for time-kill kinetics assay. Yeast cells (2×10^5^ cells/ml) were treated with 10, 20 or 30 mg/ml GHGX water-extract for 0-4 h at 37 °C in Lee’s glucose medium **(A)** or at 30 °C in YPD medium **(B)**. **(C)** Killing of *C. albicans* filamentous cells by CHGX water-extract. SC5314 filamentous cells were initially cultured in liquid Lee’s glucose medium supplemented with 20% FBS (at logarithmic phase). Filamentous cells (2×10^5^ cells/ml) were harvested and treated with 10, 20 or 30 mg/ml GHGX water-extract at 30 °C in liquid Lee’s glucose medium. The percentage of viable cells was determined using plating assays. Three biological repeats were performed, and the values are presented as mean ± SD. The Student’s *t* test (two tailed) was used to compare differences between 10 mg/ml CHGX water-extract treated samples and 20 or 30 mg/ml CHGX water-extract treated samples as indicated; *, *P* < 0.05.


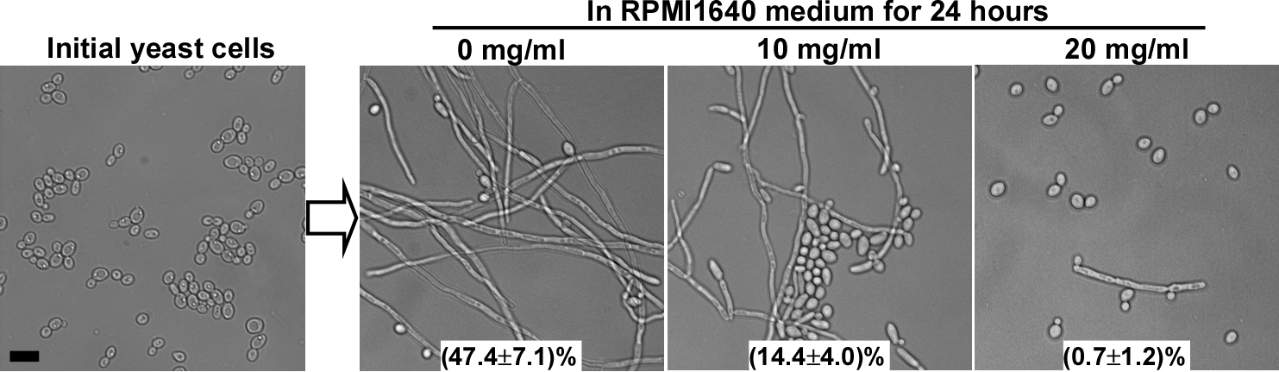


**Supplementary Figure 2. The effect of CHGX on yeast-to-hyphal transition by *C. albicans* in RPMI 1640 medium.** Initial Yeast cells of *C. albicans* (SC5314, 2×10^6^ cells/ml) were treated with 0, 10 or 20 mg/ml CHGX water-extract at 37 °C in RPMI1640 medium for 24 hours. The cellular morphology and the percentage of hyphal cells were determined by microscopic observation. Three biological repeats were performed (scale bar, 10 μm).

**Supplementary Table 1 Identification of dominant compounds of CHGX formula.**

| **No.** | ***t*_R_/min** | **Identifation** | **Molecular formula** | **Theoretical mass (Da)** | **Error (ppm)** | **ESI–MS_n_ *m/z*** | **Ref.** |
| --- | --- | --- | --- | --- | --- | --- | --- |
| 1 | 1.82 | Gallic acid | C_7_H_6_O_5_ | 169.0136[M-H]^-^ | 2.841 | 125.0235, 97.0284 | 1 |
| 2 | 3.41 | Protocatechuic acid | C_7_H_6_O_4_ | 153.0185[M-H]^-^ | 1.992 | 109.0285 | 1 |
| 3 | 5.35  5.33 | Catechin | C_15_H_14_O_6_ | 291.0861[M+H]^+^  289.0718[M-H]^-^ | -0.875  3.962 | 139.0390, 123.0442  245.0818, 123.0441, 109.0284 | 1 |
| 4 | 6.29  6.30 | Epicatechin | C_15_H_14_O_6_ | 291.0861[M+H]^+^  289.0718[M-H]^-^ | -0.772  3.962 | 139.0390, 123.0442  245.0820, 123.0441, 109.0284 | 2 |
| 5 | 8.39  8.41 | Liquiritin | C_21_H_22_O_9_ | 419.1333[M+H]^+^  417.1194[M-H]^-^ | -0.856  3.384 | 257.0808, 147.0441, 137.0234  255.0665, 153.0185, 135.0078, 119.0492 | 3 |
| 6 | 9.05  9.07 | Isoliquiritin | C_21_H_22_O_9_ | 419.1334[M+H]^+^  417.1193[M-H]^-^ | -0.713  3.072 | 257.0808, 147.0441, 137.0234  255.0665, 153.0186, 135.0079, 119.0492 | 3 |
| 7 | 9.28 | Coumarin | C_9_H_8_O_2_ | 147.0441[M+H]^+^ | 0.163 | 103.0546, 91.0547 | 3 |
| 8 | 11.11  11.10 | Ononin | C_22_H_22_O_9_ | 431.1335[M+H]^+^  475.1248[M+COOH]^-^ | -0.345  2.741 | 269.0808, 254.0571  267.0666, 252.0430 | 4 |
| 9 | 16.54 | Glycyrrhizic acid | C_42_H_62_O_16_ | 821.3970[M-H]^-^ | 1.970 | 351.0574, 193.0349, 113.0233 | 5 |
| 10 | 17.37 | Rheic acid | C_15_H_8_O_6_ | 283.0249[M-H]^-^ | 4.189 | 257.0457, 239.0350, 211.0397, 183.0446 | 6 |
| 11 | 17.89  17.91 | Pogostone | C_12_ H_16_O_4_ | 225.1122[M+H]^+^  223.0973[M-H]^-^ | 0.153  1.238 | 207.1016, 139.0390, 127.0391, 81.0704  179.1071 139.0755 | 7 |
| 12 | 18.60 | Emodin | C_15_ H_10_O_5_ | 269.0458[M-H]^-^ | 4.944 | 241.0507, 225.0556 | 6 |

**Supplementary Table 2. Strains used in this study**

| Strain | Parent strain | Genotype | Ref. |
| --- | --- | --- | --- |
| SC5314 |  | Clinical isolate | 8 |
| CAI4 | SC5314 | *MTL****a****/α ura3::imm434/ura3::imm434* | 8 |
| P37005 |  | Clinical isolate | 9 |
| CAR (G5) |  | Clinical isolate | 10 |
| 17# |  | Clinical isolate | 11 |
| BMW00716 |  | Clinical isolate | 12 |
| *C. dubliniensis* (D172) |  | Clinical isolate | 13 |
| *C. tropicalis* (JX1002) |  | Clinical isolate | This study |
| *C. auris* (BJCA001) |  | Clinical isolate | 14 |
| SN152 | CAI4 | *As CAI4, but URA3/ura3::imm434 iro1::IRO1/iro1::imm434 his1::hisG/his1::hisG leu2/leu2 arg4/arg4* | 15 |
| *ras1/ras1* | CAI4 | *MTL****a****/*α *ura3::imm434/ura3::imm434, iro1/iro1::imm434, ras1::FTR/ras1::FTR* | 16 |
| *cyr1/cyr1* | CAI4 | *MTL****a****/α cyr1::FTR/cyr1::FTR* | 16 |
| *tpk1/tpk1* | SN152 | *As SN152,* tpk1::HIS1/ tpk1::ARG4 | 17 |
| *tpk2/tpk2* | SN152 | *As SN152,* tpk2::HIS1/ tpk2::ARG4 | 17 |
| *mcu1/mcu1* | BWP17 | *ura3::imm434/URA3 his1::hisG/his1::hisG arg4::hisG/arg4::hisG mcu1::ARG4/mcu1::HIS1* | 18 |
| *Candida krusei ATCC 6258* |  | Quality control strains | This study |
| *Candida parapsilosis ATCC 22019* |  | Quality control strains | This study |

**Supplementary Table 3. Primers used in this study**

| **Name** | **Sequence (5’ to 3’)** | **Purpose** |
| --- | --- | --- |
| ACT1-F | TAAGATTATTGCTCCACCAG | *ACT1*  Q-RT-PCR |
| ACT1-R | ACCAGATTCGTCGTATTCTTG |  |
| RAS1-F | ATCAAGATGGATTAGCATTGG | *RAS1*  Q-RT-PCR |
| RAS1-R | TGTTGTTGCTGTTGTTGTTG |  |
| CYR1-F | AGAAAGAAGACGATGAAACAG | *CYR1*  Q-RT-PCR |
| CYR1-R | AGGAGAACTAGAGGATGTAGAC |  |
| TPK1-F | AGAACTTGCCAACAAACAAC | *TPK1*  Q-RT-PCR |
| TPK1-R | TTTCTTGGTCAAGGAAAGAC |  |
| TPK2-F | TTGTTGCCTGAACGTTCTAC | *TPK2*  Q-RT-PCR |
| TPK2-R | CTACCATTGTGAACTGATCTC |  |

**References**

1. Chen, L., Qi, J., Chang, Y. X., Zhu, D. and Yu, B. (2009). Identification and determination of the major constituents in Traditional Chinese Medicinal formula Danggui-Shaoyao-San by HPLC-DAD-ESI-MS/MS. J Pharm Biomed Anal. 50(2): 127-137. doi: 10.1016/j.jpba.2009.03.039.

2. Chang, C. L., Wu, R. T. (2011). Quantification of (+)-catechin and -epicatechin in coconut water by LC–MS. Food Chem. 126(2):710-717. doi: 10.1016/j.foodchem.2010.11.034.

3. Yuan, Q., Zhang, H., Zhang, Q., Wen, H., Cui, X., Peng, G., et al. (2020). Chemical Profile Analysis of Ling-Gui-Zhu-Gan Decoction by LC–QTOF MS and Simultaneous Determination of Nine Major Components Using QAMS Method. Chromatographia. 83(11):1371-1389. doi:10.1007/s10337-020-03959-z.

4. Fan, R., Li, N., Jiang, X., Yuan, F., Gao, Y. (2015). HPLC–DAD–MS/MS identification and HPLC–ABTS•+ on-line antioxidant activity evaluation of bioactive compounds in liquorice (Glycyrrhiza uralensis Fisch.) extract. Eur Food Res Technol. 240(5): 1035-1048. doi:10.1007/s00217-014-2407-5.

5. Montoro, P., Maldini, M., Russo, M., Postorino, S., Piacente, S. and Pizza, C. (2011). Metabolic profiling of roots of liquorice (Glycyrrhiza glabra) from different geographical areas by ESI/MS/MS and determination of major metabolites by LC-ESI/MS and LC-ESI/MS/MS. J Pharm Biomed Anal. 54(3): 535-544. doi: 10.1016/j.jpba.2010.10.004.

6. Hou, M. L., Chang, L. W., Lin, C. H., Lin, L. C. and Tsai, T. H. (2014). Determination of bioactive components in Chinese herbal formulae and pharmacokinetics of rhein in rats by UPLC-MS/MS. Molecules. 19(4): 4058-4075. doi: 10.3390/molecules19044058.

7. Li, Y., Su, Z., Lin, S., Li, C., Ya, Z., Gao, X., et al. (2014). Characterisation of the metabolism of pogostone in vitro and in vivo using liquid chromatography with mass spectrometry. Phytochem Anal. 25(2): 97-105. doi: 10.1002/pca.2471.

8. Fonzi, W. A. and Irwin, M. Y. (1993). Isogenic strain construction and gene mapping in Candida albicans. Genetics. 134(3): 717-728. doi: 10.1093/genetics/134.3.717.

9. Pujol, C., Pfaller, M. and Soll, D. R. (2002). Ca3 fingerprinting of Candida albicans bloodstream isolates from the United States, Canada, South America, and Europe reveals a European clade. J Clin Microbiol. 40(8): 2729-2740. doi: 10.1128/jcm.40.8.2729-2740.2002.

10. Franz, R., Kelly, S. L., Lamb, D. C., Kelly, D. E., Ruhnke, M. and Morschhäuser, J. (1998). Multiple molecular mechanisms contribute to a stepwise development of fluconazole resistance in clinical Candida albicans strains. Antimicrob Agents Chemother. 42(12): 3065-3072. doi: 10.1128/aac.42.12.3065.

11. White, T. C. (1997). Increased mRNA levels of ERG16, CDR, and MDR1 correlate with increases in azole resistance in Candida albicans isolates from a patient infected with human immunodeficiency virus. Antimicrob Agents Chemother. 41(7): 1482-1487. doi: 10.1128/aac.41.7.1482.

12. Gong, J., Wu, J., Ikeh, M., Tao, L., Zhang, Y., Bing, J., et al. (2019). Antifungal Activity of Mammalian Serum Amyloid A1 against Candida albicans. Antimirobial Agents and Chemotherapy. 64(1). doi: 10.1128/aac.01975-19.

13. Joly, S., Pujol, C., Rysz, M., Vargas, K. and Soll, D. R. (1999). Development and characterization of complex DNA fingerprinting probes for the infectious yeast Candida dubliniensis. J Clin Microbiol. 37(4): 1035-1044. doi: 10.1128/jcm.37.4.1035-1044.1999.

14. Wang, X., Bing, J., Zheng, Q., Zhang, F., Liu, J., Yue, H., et al. (2018). The first isolate of Candida auris in China: clinical and biological aspects. Emerg Microbes Infect. 7(1): 93. doi: 10.1038/s41426-018-0095-0.

15. Noble, S. M. and Johnson, A. D. (2005). Strains and strategies for large-scale gene deletion studies of the diploid human fungal pathogen Candida albicans. Eukaryotic cell. 4(2): 298-309.

16. Yi, S., Sahni, N., Daniels, K. J., Lu, K. L., Srikantha, T., Huang, G., et al. (2011). Alternative mating type configurations (a/α versus a/a or α/α) of Candida albicans result in alternative biofilms regulated by different pathways. PLoS Biol. 9(8): e1001117. doi: 10.1371/journal.pbio.1001117.

17. Cao, C., Wu, M., Bing, J., Tao, L., Ding, X., Liu, X., et al. (2017). Global regulatory roles of the cAMP/PKA pathway revealed by phenotypic, transcriptomic and phosphoproteomic analyses in a null mutant of the PKA catalytic subunit in Candida albicans. Molecular Microbiology. 105(1): 46-64. doi: 10.1111/mmi.13681.

18. Guan, G., Wang, H., Liang, W., Cao, C., Tao, L., Naseem, S., et al. (2015). The mitochondrial protein Mcu1 plays important roles in carbon source utilization, filamentation, and virulence in Candida albicans. Fungal Genet Biol. 81: 150-159. doi: 10.1016/j.fgb.2015.01.006.
